# Effect of Village-wide Use of Long-Lasting Insecticidal Nets on Visceral Leishmaniasis Vectors in India and Nepal: A Cluster Randomized Trial

**DOI:** 10.1371/journal.pntd.0000587

**Published:** 2010-01-26

**Authors:** Albert Picado, Murari L. Das, Vijay Kumar, Shreekant Kesari, Diwakar S. Dinesh, Lalita Roy, Suman Rijal, Pradeep Das, Mark Rowland, Shyam Sundar, Marc Coosemans, Marleen Boelaert, Clive R. Davies

**Affiliations:** 1 London School of Hygiene and Tropical Medicine, London, United Kingdom; 2 B. P. Koirala Institute of Health Sciences, Dharan, Nepal; 3 Rajendra Memorial Research Institute of Medical Sciences, Patna, India; 4 Banaras Hindu University, Varanasi, India; 5 Prince Leopold Institute of Tropical Medicine, Antwerp, Belgium; Johns Hopkins Bloomberg School of Public Health, United States of America

## Abstract

**Background:**

Visceral leishmaniasis (VL) control in the Indian subcontinent is currently based on case detection and treatment, and on vector control using indoor residual spraying (IRS). The use of long-lasting insecticidal nets (LN) has been postulated as an alternative or complement to IRS. Here we tested the impact of comprehensive distribution of LN on the density of *Phlebotomus argentipes* in VL-endemic villages.

**Methods:**

A cluster-randomized controlled trial with household *P. argentipes* density as outcome was designed. Twelve clusters from an ongoing LN clinical trial—three intervention and three control clusters in both India and Nepal—were selected on the basis of accessibility and VL incidence. Ten houses per cluster selected on the basis of high pre-intervention *P. argentipes* density were monitored monthly for 12 months after distribution of LN using CDC light traps (LT) and mouth aspiration methods. Ten cattle sheds per cluster were also monitored by aspiration.

**Findings:**

A random effect linear regression model showed that the cluster-wide distribution of LNs significantly reduced the *P. argentipes* density/house by 24.9% (95% CI 1.80%–42.5%) as measured by means of LTs.

**Interpretation:**

The ongoing clinical trial, designed to measure the impact of LNs on VL incidence, will confirm whether LNs should be adopted as a control strategy in the regional VL elimination programs. The entomological evidence described here provides some evidence that LNs could be usefully deployed as part of the VL control program.

**Trial registration:**

ClinicalTrials.gov CT-2005-015374

## Author Summary

Visceral leishmaniasis (VL) is a vector-borne disease causing at least 60,000 deaths each year amongst an estimated half million cases, and until recently there have been no significant initiatives to reduce this burden. However, in 2005, the governments of India, Bangladesh and Nepal signed a memorandum of understanding at the World Health Assembly in Geneva for the elimination of the disease by 2015. In the absence of an effective vaccine, the program will rely on the active detection and prompt treatment of cases throughout the endemic region, combined with a recurrent indoor residual spraying (IRS) of all villages at risk. Vector control programs based on IRS are notorious for failing to maintain comprehensive spray coverage over time owing to logistical problems and lack of compliance by householders. Long-lasting insecticidal nets (LNs) have been postulated as an alternative or complement to IRS. Here we describe how comprehensive coverage of LN in trial communities reduced the indoor density of sand flies by 25% compared to communities without LNs. This provides an indication that LNs could be usefully deployed as a component of the VL control program in the Indian subcontinent.

## Introduction

Visceral leishmaniasis (VL), also known as kala azar, is a life-threatening vector-borne disease with a fatal outcome if left untreated. A large proportion of the 500,000 annual cases and 60,000 deaths occur in the poor rural communities within the Indian subcontinent [1]. In this region, VL is caused by *Leishmania donovani* and is transmitted by the sand fly *Phlebotomus argentipes* [2]. Elimination of VL in the region is deemed feasible because humans are the only known reservoir and VL is confined to contiguous areas within the Indian subcontinent. In the absence of a human leishmaniasis vaccine, the elimination program proposes to reduce VL incidence by active case detection and treatment, and the widespread use of residual insecticide spraying (IRS) of houses and cattle sheds with DDT in India and with pyrethroids in Nepal and Bangladesh. Public health policy makers in the region are aware that long-term house-spraying campaigns against VL vectors may also be difficult to sustain [3]–[5]. Hence, it has been suggested that, as for malaria control in India, it may be more efficient to provide insecticide treated nets (ITNs) to the population at risk for VL [5]. Given the well known difficulties in maintaining high re-impregnation rates of ITNs, malaria vector control programs are now implementing WHOPES-recommended long-lasting insecticidal nets (LNs), such as PermaNet 2.0 and Olyset.

There is no clear evidence that use of ITNs would reduce VL incidence in the Indian subcontinent. However, circumstantial evidence drawn from *P. argentipes* behavioral studies provides hope that ITN will work. In particular *P. argentipes* is relatively endophagic [6] and indoor biting rhythms peaks during the middle of the night when people are in bed [6]–[8].

Evidence that village-wide use of ITNs can protect against other forms of leishmaniasis transmitted by a variety of sand fly species come from several small scale trials in Iran [9]–[11], Sudan [12], Syria [13] and Turkey [14] and two large scale trials in Iran [15] and Syria [16]. There is also encouraging circumstantial evidence from a retrospective evaluation of a large ITN VL control program in Sudan [17].

Studies on malaria vector control have provided evidence that ITNs, not only provide personal protection to the user, but can, in certain situations, such as when coverage is high enough, provide protection to the entire community, non users included, through the reduction of vector population density [18]–[20]. Until now there has been no reported evidence that community-wide use of ITNs can generate a similar effect against any form of leishmaniasis infection. Most entomological studies, such as the trials in Iran [9]–[11], Syria [13] and Turkey [14], have singularly failed to demonstrate any effect on sand fly density through community-wide use of ITNs. The only evidence we are aware of is an Iranian trial which detected a reduction in sand fly (*P. sergenti*) density in the ITN villages [15] and a Sudanese trial in which infections caused by *P. orientalis* may have been reduced in villages using ITNs although the results were non-statistically significant [21]. It appears that community-wide ITN usage is more likely to reduce sand fly density and infection rate if a large proportion of sand flies feed on humans and if the leishmania is anthroponotic. Both criteria are true for VL transmitted by *P. argentipes* [22]–[24].

The objective of this study was to assess the effect of comprehensive coverage of LNs in VL endemic villages in Nepal and India on *P. argentipes* indoor density, with the expectation that a major effect would have implications for future vector control strategy adopted by the regional VL elimination program.

## Methods

### Study area

**Figure 1:**
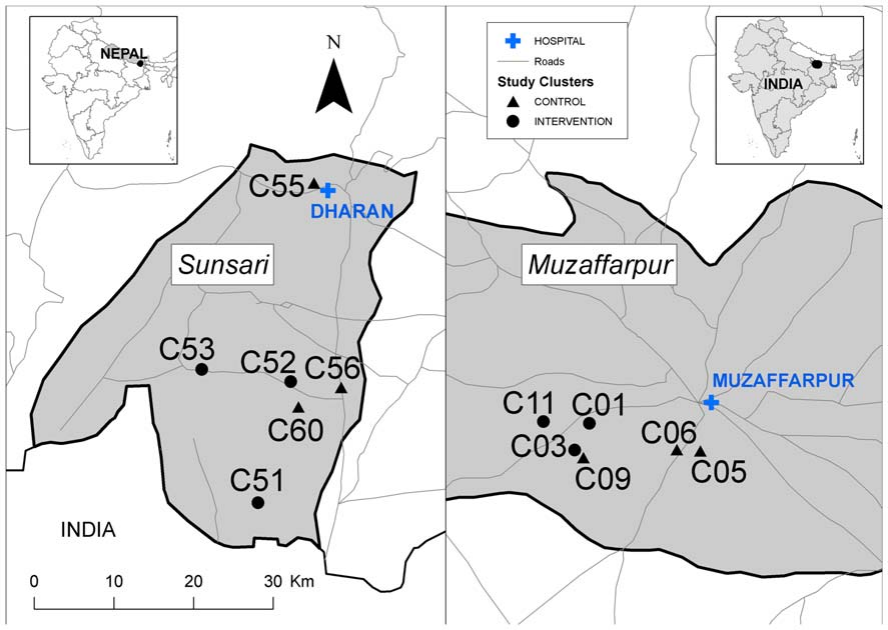
Location of the 12 clusters, 6 in Muzaffarpur district, India and 6 in Sunsari district, Nepal selected for the entomological trial. Intervention and control clusters are identified with a circle and a triangle respectively.

The study area is comprised of two field sites from the VL endemic region in the Indian subcontinent with 6 clusters in Muzaffarpur district, India and 6 in Sunsari district, Nepal (Figure 1). Most clusters were complete villages but others were easily identifiable administrative subdivisions (i.e. ward) of larger populated areas. All clusters were endemic for VL and share similar ecologic and climatologic conditions. Eleven of the clusters were rural and one was peri-urban. The use of untreated bed nets was common with 46% and 64% of households in India and Nepal, respectively, owning at least one bed net before the trial started. Some of the villages were sprayed as a part of the national VL control programs of India and Nepal using DDT and alpha-cypermethrin, respectively. For ethical reasons there was no interference in the execution of these programs but IRS information was gathered and considered in the analysis.

### Study design

#### Selection and randomization of KALANET clusters

The 12 clusters included in this entomological trial are a subset of the 26 clusters selected for the ongoing KALANET trial (ClinicalTrials.gov CT-2005-015374). The KALANET study has two components: (1) a 2-year follow up clinical trial designed to detect a 50% reduction in *L. donovani* incidence rates in the 13 LN intervention clusters compared to the 13 matched control clusters; and (2) a 12-month follow up entomological trial to measure the impact of LNs on *P. argentipes* indoor density in 6 intervention and 6 control clusters. There were two main outcomes for the clinical trial; the number of individuals seroconverting and the number of confirmed incident VL cases in intervention and control groups. In this paper we present the results from the entomological trial using the *P. argentipes* indoor density as the outcome. Measuring the impact of LNs on sand fly vector population supports the interpretation of any observed impact on clinical outcomes; and, in particular, provides an opportunity to distinguish the relative importance of LNs on personal protection as compared to community level protection.

The number of clusters included in the clinical trial was determined using sample size formulae developed for cluster randomized trials [25]. The selection of clusters is briefly described here. In February 2006, a house-to-house survey was conducted in 34 clusters (22 in India and 12 in Nepal) with historically high numbers of VL cases to estimate incidence rates from 2003 to 2005. The final list of 26 clusters (16 in India and 10 in Nepal) was selected according to four criteria: (1) new cases of VL must have been recorded during each of the previous three years, (2) the average VL incidence rate for the past three years should have exceded 0.8%, (3) cluster populations of between 350 and 1500 people, (4) a minimum distance of 1 km between any two clusters. Clusters fulfilling all of these criteria were selected according to the highest incidence rates.

The 26 clusters were stratified by country, population size and then paired by VL incidence rates. Clusters in each pair were randomly allocated to LN or control groups. To maximize the chances of obtaining comparable groups at baseline, a simple constraint was applied: the difference in the total number of VL cases reported in the last 3 years between group 1 and 2 must be less than 10%.

#### Selection of entomological trial clusters

Out of the 26 KALANET clusters, 3 intervention and 3 control clusters in each country were selected for the entomological trial on the basis of year round accessibility and VL incidence rates. 13 clusters were initially assessed (6 in India and 7 in Nepal) and one was finally excluded in Nepal. Being a subset of the KALANET clusters, the 12 selected clusters for the entomological trial were not necessarily paired.

The objective of the study was to detect a significant reduction of the number of *P. argentipes*/house in the LN clusters compared to the control clusters. Power calculations for the entomological trial were not carried out because there were no data on either the mean or variance, within or between clusters, in the expected number of *P. argentipes* per house over 12 months. Instead, we elected to monitor the maximum number of clusters and houses per cluster (see below) logistically feasible with the available resources. The power of the study was also maximized by selecting, where possible, those clusters where highest sand fly densities were expected on the basis of reported VL data.

#### Selection of households

In September 2006, twenty five households per cluster were randomly selected, with the proviso that none of the selected houses could be on the edge of the cluster. The 10 households in each cluster with the highest number of *P. argentipes*, as determined by aspiration, were then recruited into the entomological trial. Ten houses per cluster per month was the maximum feasible within the logistic constraints of personnel and equipment. Statistical power was maximized by deliberately targeting those houses with highest *P. argentipes* density. Ten cattle sheds per cluster (sheds from the selected or nearest houses) were also selected for entomological monitoring (inclusion criteria: a roof, a wall and no connection with living quarters).

Written informed consent for recruitment into the trial was obtained from the head of the households. A literate witness signed on behalf of illiterate participants who added their thumbprint to the informed consent form.

The research ethics committees of the Indian Council of Medical Research in India, the B. P. Koirala Institute of Health Sciences in Nepal, the Prince Leopold Institute of Tropical Medicine in Belgium and the London School of Hygiene and Tropical Medicine in the UK reviewed and approved this study.

#### Entomological monitoring and intervention

Monthly entomological surveys were carried out in each cluster from September 2006 to November and December 2007 in India and Nepal respectively using CDC Light traps (LT) set up in the 10 selected houses 1 night per month from 6pm to 6am. Light traps were positioned in the corner of an occupied bedroom 1 inch from the wall and 6 inches from the floor. The following morning between 6 and 9am sand flies were also collected in the same houses by aspiration for 15 minutes per room and 15 minutes per cattle shed. Collected sand flies were identified under a binocular microscope to species for *Phlebotomus* and to genus for *Sergentomyia*. Gender and presence of blood were also recorded. Mosquitoes collected in light traps were recorded to subfamily and gender, species identification being beyond the scope of this project which was primarily designed to measure impact of bed nets on VL vectors.

Pre-intervention collections for 4 months in India and 3 in Nepal served as baseline to confirm that the number of sand flies collected in intervention and control groups were comparable. In both countries there were 12 monthly post-intervention collections.

LNs (PermaNet 2.0) were distributed in all households of the intervention clusters at the end of November 2006 in Nepal and at the end of December 2006 in India. Enough nets were provided to protect all household members. The control clusters did not receive bed nets until the end of the KALANET clinical trial (December 2008) but were provided with free diagnosis and treatment for VL during the trial.

To assess the effect of cluster-wide distribution of LNs on sand fly numbers in the LTs rather than to a local household effect induced by the presence of the LN during sand fly capture, i.e. due to LN-induced deterrence, excito-repellency or immediate sand fly mortality [26],[27], from the first month that LNs were provided to the trial clusters, untreated bed nets were lent to household members of sentinel houses for the night of each monthly capture to temporarily replace the LNs in regular use in intervention clusters. To maintain comparability untreated nets were also provided to the sentinel households in the control clusters on the night of LT collecting.

### Analyses

#### Data

Owing to the dispersed distribution of entomological data for both LT and aspiration collections, monthly data collections were summarized using the Williams modified geometric means (GM).

#### Effect of LNs

The effect of LNs on sand fly and mosquito densities was assessed by fitting a random effect linear regression model to the LT and aspiration data. The outcome data were the log transformed GM monthly collection for each household post-intervention data (i.e. a mean of 12 replicates/house). Intervention was treated as the principal explanatory variable in a model adjusted by country and pre-intervention log transformed GM with cluster as a random-effect to take into account the clustering of the data. The interaction term between country and intervention variables was tested to determine whether the distribution of LNs varied in the two field sites. The suitability of all models was confirmed by inspection of the distribution of residuals.

The analyses were repeated after adjusting for the possible effect of any house spraying carried out by the health ministries in the trial clusters by adding a dichotomous variable with the information on IRS at cluster level during the post-intervention period to the regression model.

All statistical analyses were performed using Stata 10 (StataCorp LP, College Station, TX, USA).

## Results

### Household flow

**Figure 2:**
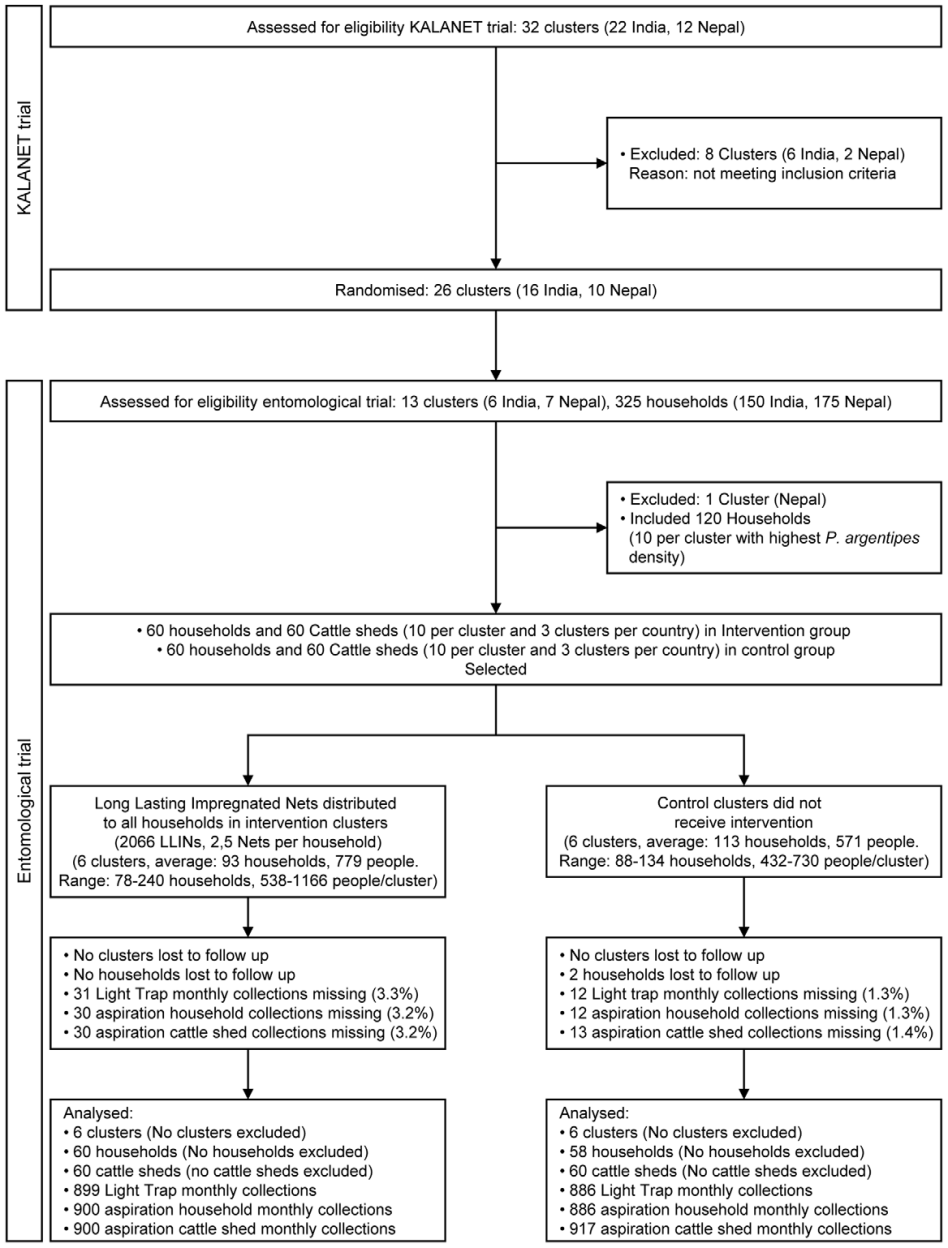
Flow diagram of clusters and households (and cattle sheds) through study. The upper part corresponds to the cluster selection for the full KALANET clinical trial and the lower part details the progress for the subset used in the entomological trial described in this paper.

As shown in Figure 2, only 2 out of 120 households initially included in the trial were lost to follow up, both in the same control cluster in India. The number of monthly collections missing was 3% in the intervention group and 1% in the control. Causes of missing collections included a faulty LT in Nepal in September 2006, 2 households which were not accessible in August 2007 in India, incidents of flooding and other logistical and accessibility problems. In each country two clusters could not be monitored during August and December 2007 for one intervention and one control cluster respectively in India and during January for two intervention clusters in Nepal.

In India only one of the clusters was not sprayed with DDT after the distribution of LNs. Between January and December 2007 one intervention and one control cluster were sprayed twice and the rest only once. In Nepal a single control cluster was sprayed twice in the same time frame.

### Sand fly captures

The sand fly fauna differed markedly between the Indian and Nepali field sites. In Nepal, a total of 12285 sand flies were collected and identified from 879 house-night LT catches (total number of sand flies, N = 7650), from 880 house aspirations (N = 3481) and from 879 cattle shed aspirations (N = 1154). Of these, only 29.3% were *P. argentipes*. The percentage was highest in the LT collections (41.1%) compared to 13.3% in aspirations from cattle sheds and 8.6% from house aspirations. The remainder were *P. papatasi* (24.6%) and *Sergentomyia spp*. (46.1%). *P. papatasi* was more represented in the house aspirations (36.2% of total) whereas *Sergentomyia spp*. was more common in the cattle shed aspirations (67.3%). In India, a total of only 2539 sand flies were collected and identified: from 938 LT catches (N = 1952), from 938 house aspirations (N = 325) and 938 cattle shed aspirations (N = 262). Of these, 94.2% were *P. argentipes*. The percentage was highest in the LT collections (94.9%) compared to 94.7% from cattle shed aspirations and 89.2% from house aspirations. The remainder were almost all *Sergentomyia spp.* (5.7%) with only 2 *P. papatasi* collected in total.

The sex ratio of *P. argentipes* collected by LT was close to 50% in both India and Nepal (56.5% and 45.9% female, respectively). But the sex ratio of resting site aspirations differed considerably between countries: 57.9% female from house and 58.9% female from cattle shed aspirations in India, compared to 80.1% and 79.9% in the respective collections in Nepal. In both countries, the percentage of blood fed *P. argentipes* was higher in the aspiration than the LT collections. In India, the percentage blood-fed were 20.8% in house and 32.9% in cattle shed aspirations, compared to 14.8% in the LT collections. In Nepal the rates were 56.0%, 43.9% and 4.7%, respectively.

### Effect of LNs on *P. argentipes*

Table 1 is a summary of all pre-intervention *P. argentipes* collected by LT and mouth aspirator in households grouped by cluster, country and intervention group. The baseline analyses confirmed that the control and LN clusters were comparable according to *P. argentipes* LT total GM densities (p = 0.931) and by *P. argentipes* LT GM female densities (p = 0.298). The same was true for analysis of pre-intervention density based on aspiration.

**Table 1:** Summary of all *P. argentipes* collected (totals and Williams modified Geometric means) in households by light trap (LT) and mouth aspirator during the pre-intervention period (September – November 2006 in Nepal and September – December 2006 in India), according to gender and blood meal status.

Cluster	*CDC Light Traps*				*Mouth aspirator*			
	LT nights	Total (females)	GM total (GM females)	Blood fed (%)	Aspirations	Total (females)	GM total (GM females)	Blood fed (%)
***Intervention group***								
C01	40	114 (61)	2.2 (1.2)	9 (14.8)	40	29 (17)	0.4 (0.26)	10 (58.8)
C03	40	135 (58)	2.2 (1.1)	13 (22.4)	40	29 (18)	0.34 (0.26)	9 (50)
C11	40	161(88)	2.8 (1.5)	35 (39.8)	40	21 (10)	0.3 (0.14)	2 (20)
C51	30	356 (174)	6.0 (2.7)	11 (6.3)	30	21 (18)	0.3 (0.29)	10 (55.6)
C52	30	147 (39)	2.8 (0.9)	3 (7.7)	30	23 (18)	0.35 (0.28)	13 (72.2)
C53	29	88 (36)	1.5 (0.7)	1 (2.8)	30	16 (13)	0.29 (0.25)	6 (46.2)
**Total**	**209**	**1001 (456)**	**2.9 (1.3)**	**72 (15.8)**	**210**	**139 (94)**	**0.33 (0.24)**	**50 (53.2)**
***Control group***								
C05	40	121 (83)	2.2 (1.5)	22 (26.5)	40	36 (29)	0.39 (0.34)	5 (17.2)
C06	40	146 (85)	2.5 (1.5)	41 (48.2)	40	32 (19)	0.41 (0.26)	4 (21.1)
C09	32	211 (113)	3.2 (1.8)	22 (19.5)	32	28 (12)	0.32 (0.16)	2 (16.7)
C55	30	82 (49)	1.3 (0.9)	6 (12.2)	30	13 (9)	0.23 (0.18)	2 (22.2)
C56	30	277 (117)	3.8 (1.7)	16 (13.7)	30	21 (19)	0.22 (0.19)	12 (63.2)
C60	30	208 (83)	4.8 (1.8)	7 (8.4)	30	16 (13)	0.29 (0.26)	10 (76.9)
**Total**	**202**	**1045 (530)**	**3.0 (1.5)**	**114 (21.5)**	**202**	**146 (101)**	**0.32 (0.24)**	**35 (34.7)**

Data presented by cluster and stratified by intervention grouping (long lasting insecticidal nets or control). Cluster codes are KALANET clinical trial codes: C01 to C11 and C51 to C60 clusters from India and Nepal respectively.

**Figure 3:**
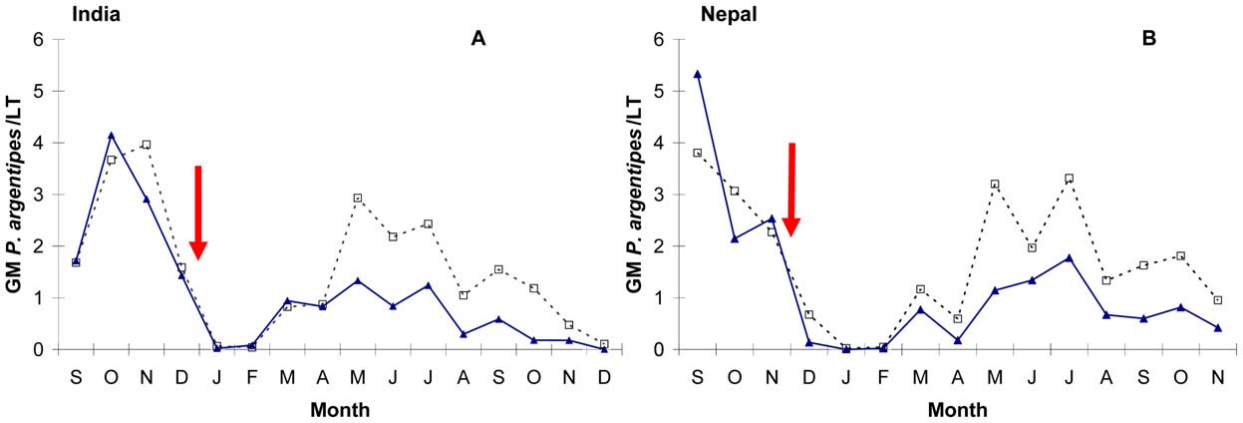
Monthly geometric mean (GM) of total *P. argentipes/*LT/house for intervention (solid line) and control (dotted line) clusters. (A) India and (B) Nepal. The arrow indicates the time of intervention (distribution of long lasting insecticidal nets).

Figure 3 presents the monthly GM of total *P. argentipes* in intervention and control groups in India and Nepal. The number of *P. argentipes* significantly drops in both control and intervention clusters between 2006 and 2007. However from May 2007, the reduction of *P. argentipes* density in the LN group is consistently higher than in control. Table 2 summarizes the number of *P. argentipes* trapped by LT and collected by aspiration in houses during 12-month post-intervention by cluster, country and intervention group. The principal finding from the analyses at a household level was that the cluster-wide distribution of LNs significantly reduced the 12-month post-intervention GM of total *P. argentipes*/house by 24.9% (95% CI 1.80–42.5%), p = 0.036 in intervention compared to control clusters. The effect was not significantly different in the two countries (interaction term: p = 0.953). However, the adjustment for Ministry of Health spraying activity caused an increase in the estimated impact of LNs on total *P. argentipes* to 31.9% (95% CI 12.6–46.9%), p = 0.003. This was exclusively due to the impact of spraying in Nepal, the Indian spraying activity having no detectable impact on our estimation of the effect of cluster-wide distribution of LNs. The analyses carried out on *P. argentipes* females also found a significant reduction in GM density of 11.6% (95% CI 2.10–20.2%), p = 0.016. The analysis based on aspiration collections in houses did not show any significant effect of LNs on *P. argentipes* abundance (reduction 3.3%; 95% CI −3.9–10.1%; p = 0.356).

**Table 2:** Summary of all *P. argentipes* collected (totals and Williams modified Geometric means) in households by light trap (LT) and mouth aspirator during the 12-month post-intervention (December 06 to November 07 in Nepal and January–December 07 in India) according to gender and blood meal status.

Cluster	*CDC Light Traps*				*Mouth aspirator*			
	LT nights	Total (females)	GM total (GM females)	Blood fed (%)	Aspirations	Total (females)	GM total (GM females)	Blood fed (%)
***Intervention group***								
C01	110	90 (59)	0.5 (0.3)	1 (1.7)	110	6 (3)	0.04 (0.02)	0 (0)
C03	120	94 (54)	0.5 (0.3)	0 (0.0)	120	10 (5)	0.05 (0.03)	0 (0)
C11	120	100 (54)	0.5 (0.3)	1 (1.9)	120	16 (9)	0.08 (0.05)	0 (0)
C51	110	308 (153)	1.4 (0.7)	0 (0.0)	110	17 (14)	0.08 (0.07)	9 (64.3)
C52	110	81 (38)	0.4 (0.2)	1 (2.6)	110	58 (44)	0.23 (0.2)	30 (68.2)
C53	120	50(20)	0.3 (0.1)	2 (10.0)	120	21 (16)	0.08 (0.07)	8 (50)
**Total**	**690**	**723 (378)**	**0.6 (0.3)**	**5 (1.3)**	**690**	**128 (91)**	**0.09 (0.07)**	**47 (51.6)**
***Control group***								
C05	110	200 (121)	1.1 (0.7)	2 (1.6)	110	27 (12)	0.14 (0.07)	1 (8.3)
C06	118	185 (112)	0.8 (0.5)	1 (0.9)	118	21 (12)	0.1 (0.06)	0 (0)
C09	96	186 (106)	1.1 (0.7)	5 (4.7)	96	29 (19)	0.17 (0.12)	1 (5.3)
C55	120	154 (75)	0.6 (0.3)	1 (1.3)	120	33 (30)	0.11 (0.1)	21 (70)
C56	120	1209 (554)	2.8 (1.4)	19 (3.4)	120	50 (39)	0.18 (0.15)	11 (28.2)
C60	120	184 (106)	0.7 (0.4)	1 (0.9)	120	12 (8)	0.06 (0.04)	3 (37.5)
**Total**	**684**	**2118 (1074)**	**1.2 (0.7)**	**29 (2.7)**	**684**	**172 (120)**	**0.13 (0.09)**	**37 (30.8)**

Data presented by cluster and stratified by intervention grouping (long lasting insecticidal nets or control). Cluster codes are KALANET clinical trial codes: C01 to C11 and C51 to C60 clusters from India and Nepal respectively.

All 12-month post-intervention *P. argentipes* collected by mouth aspirator in cattle sheds are summarized by cluster and intervention group in table 3 (pre-intervention captures information is available as additional material). The analysis of the cattle shed data did not show any significant reduction of *P. argentipes* density in cattle sheds in intervention clusters compared to control (reduction 0.7%; 95% CI −6.1–7.1%; p = 0.838).

**Table 3:** Summary of all *P. argentipes* collected (totals and Williams modified Geometric means) in cattle sheds by mouth aspirator during the 12-month post-intervention (December 06 to November 07 in Nepal and January–December 07 in India) according to gender.

Cluster	Aspirations	Total (females)	GM total (GM females)
***Intervention group***			
C01	110	8 (6)	0.04 (0.03)
C03	120	25 (15)	0.12 (0.08)
C11	120	20 (14)	0.09 (0.07)
C51	110	68 (56)	0.28 (0.24)
C52	110	0 (0)	0 (0)
C53	120	4 (3)	0.02 (0.01)
**Total**	**690**	**125 (94)**	**0.09 (0.07)**
***Control group***			
C05	110	15 (10)	0.07 (0.05)
C06	118	22 (16)	0.09 (0.06)
C09	120	29 (19)	0.13 (0.1)
C55	119	2 (1)	0.01 (0.01)
C56	120	41 (32)	0.16 (0.13)
C60	120	0 (0)	0 (0)
**Total**	**707**	**109 (78)**	**0.08 (0.06)**

Data presented by cluster and stratified by intervention grouping (long lasting insecticidal nets or control). Cluster codes are KALANET clinical trial codes: C01 to C11 and C51 to C60 clusters from India and Nepal respectively.

The effect of LN on blood feeding rates could not be tested due to the low number of blood fed *P. argentipes* collected post-intervention (91 flies in intervention and 120 in control clusters). Blood feeding rates per cluster, country and intervention group collected in households by LTs and aspiration pre and post-intervention are presented in tables 1 and 2 respectively.

### Effect of LN on *P. papatasi* and mosquitoes

No reduction in *P. papatasi* LT collections was detected in Nepal due to LNs (reduction −13.0%; 95% CI −59.3–19.9%; p = 0.486). No analyses were carried out in India as only one *P. papatasi* was collected using LTs in India.

Mosquito abundance was much higher in the Nepali field sites than the Indian sites, with a total of 60214 culicine and 5535 anopheline mosquitoes collected in Nepal compared to only 4245 and 5205, respectively, in India. The number of culicine and anopheline post-intervention per cluster and intervention group are summarized in table 4. The 12-month post-intervention GM of total anopheline density was statistically reduced with an average reduction of 22.9% (95% CI 12.9–31.8%), p<0.001 as shown by the regression model. In contrast, culicine densities were not reduced in intervention clusters compared to control (reduction −4.0%; 95% CI −48.5–27.2%; p = 0.829).

**Table 4:** Summary of all *P. papatasi*, culicine and anopheline collected (totals and Williams modified Geometric means) in households by light trap (LT) during the 12-month post-intervention (December 06 to November 07 in Nepal and January–December 07 in India) according to gender.

Cluster		*P. Papatasi*		Culicine		Anopheline	
	LT nights	Total (females)	GM total (GM females)	Total (females)	GM total (GM females)	Total (females)	GM total (GM females)
***Intervention group***							
C01	110	0 (0)	0 (0)	416 (238)	2.6 (1.6)	467 (227)	2.6 (1.3)
C03	120	0 (0)	0 (0)	514 (304)	3.2 (2.0)	582 (313)	3.4 (1.9)
C11	120	0 (0)	0 (0)	580 (323)	3.4 (2.0)	583 (291)	3.3 (1.8)
C51	110	328 (154)	1.01 (0.66)	14736 (13592)	42.0 (37.1)	1129 (1111)	2.0 (1.8)
C53	120	174 (82)	0.57 (0.31)	6953 (6369)	32.1 (29.2)	247 (205)	1.1 (0.9)
C52	110	40 (15)	0.13 (0.07)	4468 (4071)	18.0 (16.5)	313 (249)	1.2 (1.0)
**Total**	**690**	**542 (251)**	**0.27 (0.17)**	**27667 (24897)**	**16.9 (14.7)**	**3321 (2396)**	**2.3 (1.4)**
***Control group***							
C05	110	0 (0)	0 (0)	625 (357)	4.6 (2.7)	746 (392)	5.2 (2.8)
C06	118	1 (1)	0.01 (0.01)	613 (352)	3.8 (2.3)	724 (410)	4.3 (2.5)
C09	96	0 (0)	0 (0)	564 (323)	4.8 (2.7)	637 (334)	5.0 (2.6)
C55	120	30 (7)	0.14 (0.03)	1321 (787)	4.6 (2.9)	366 (338)	0.7 (0.6)
C56	120	333 (139)	0.8 (0.45)	14611 (14049)	50.2 (46.5)	1831 (1569)	4.0 (3.6)
C60	120	24 (15)	0.11 (0.07)	4405 (4206)	13.1 (12.2)	564 (517)	1.9 (1.7)
**Total**	**684**	**388 (162)**	**0.19 (0.1)**	**22139 (20074)**	**13.5 (11.5)**	**4868 (3560)**	**3.5 (2.3)**

Data presented by cluster and stratified by intervention grouping (long lasting insecticidal nets or control). Cluster codes are KALANET clinical trial codes: C01 to C11 and C51 to C60 clusters from India and Nepal respectively.

## Discussion

The trial provided the first evidence that community-wide distribution of ITNs can reduce the indoor *P. argentipes* density in VL endemic villages. Such effects can be due to a genuine reduction in population density brought about by insecticide induce mortality or be due to local displacement of sand flies brought about by insecticide repellency. The effect here seems to be caused by reduction in the population density of *P. argentipes* at a community level rather than to displacement. This inference is supported by the observation that *P. argentipes* density in sentinel cattle sheds was not increased, as would be expected if simple re-distribution of sand flies within the cluster was the cause. However results based on aspiration captures in cattle sheds should be interpreted with caution as the number of *P. argentipes* collected was highly variable between and within clusters. This conclusion stands in contrast to a previous study where LNs given to selected households in the community did not reduce the indoor vector density [28].

The 25% reduction in sand fly density is much lower than the 59–80% reduction in mosquito density observed in malaria control trials of ITNs in Africa [19] and may have a more limited effect on *L. donovani* transmission as compared to *Plasmodium* transmission rates. Some of the factors that may explain the difference between sand fly and mosquito reductions include the lower level of anthropophagy of *P. argentipes* compared to *An. gambiae*, the time of vector biting, the extent of movement of vectors from untreated areas to treated areas and vector susceptibility to the insecticide. One or more of these factors may be the reason why the LNs in our trial caused a significant reduction in anopheline densities, but had no discernible effect on culicine densities, and reduced *P. argentipes* density but not on *P. papatasi*. The floods in the study areas during the trial may explain the decline of *P. argentipes* density between the same periods in 2006 and 2007 (Figure 3) as extreme rainfall has been related to reduction of sand fly abundance in previous studies [29].

The effect of LNs on the number of blood fed vectors in households as determined by aspiration collections may have been a better indicator on the impact of LNs on biting rates as LTs are not very effective in catching blood fed *P. argentipes* [30]. Unfortunately the low number of female *P. argentipes* captured by aspiration in households post-intervention made impossible any formal statistical analysis. The overall blood feeding rates post-intervention −52% in intervention and 31% in control groups - should be interpreted with caution as aspiration captures were clustered in only a few households (i.e. no females were collected in 52 households and 4 households concentrated 23% of the total captures).

The strategic decision by the regional VL elimination program whether to include LNs to communities at risk will depend on the findings of the ongoing KALANET clinical trial which is designed to measure the impact of LNs on VL incidence and secondly the cost effectiveness of the strategy. However, the entomological results described here provide a powerful argument that if LNs were to be incorporated into the elimination program, a high household coverage, even if the minimum threshold is unknown, would be required to generate an effect on *P. argentipes* density. The reduction due to use of LNs was however rather limited and was not enhanced, at least in India, by IRS. The current vector control programs in the region are failing to control VL [31] and LNs may prove to be an appropriate complement to IRS, improving their efficacy and sustainability. Further work on integrated vector management for *P. argentipes* should be carried out to determine the best combination of tools to effectively control *L. donovani* vector in the Indian subcontinent.
